# Shear wave elastography and dispersion imaging for hepatic veno-occlusive disease prediction after pediatric hematopoietic stem cell transplantation: a feasibility study

**DOI:** 10.1007/s00247-024-05940-6

**Published:** 2024-05-22

**Authors:** Seul Bi Lee, Seunghyun Lee, Yeon Jin Cho, Young Hun Choi, Jung-Eun Cheon, Kyung Taek Hong, Jung Yun Choi, Hyoung Jin Kang

**Affiliations:** 1 Department of Radiology, Seoul National University Hospital, 101 Daehak-ro, Jongno-gu, Seoul, 03080 Republic of Korea; 2 Department of Radiology, Seoul National University College of Medicine, Seoul, Republic of Korea; 3 Institute of Radiation Medicine, Seoul National University Medical Research Center, Seoul, Republic of Korea; 4 Department of Pediatrics, Seoul National University Hospital, Seoul, Republic of Korea; 5 Department of Pediatrics, Seoul National University College of Medicine, Seoul, Republic of Korea; 6 Seoul National University Cancer Research Institute, Seoul, Republic of Korea; 7 Wide River Institute of Immunology, Seoul National University, Hongcheon-gun, Gangwon-do Republic of Korea

**Keywords:** Child, Elasticity imaging techniques, Hematopoietic stem cell transplantation, Hepatic veno-occlusive disease, Ultrasonography

## Abstract

**Background:**

Non-invasive imaging modalities are warranted for diagnosing and monitoring veno-occlusive disease because early diagnosis and treatment improve the prognosis.

**Objective:**

To evaluate the usefulness of liver shear wave elastography (SWE) and shear wave dispersion (SWD) imaging in diagnosing and monitoring veno-occlusive disease in pediatric patients.

**Materials and methods:**

We conducted a prospective cohort study at a single tertiary hospital from March 2021 to April 2022. The study protocol included four ultrasound (US) sessions: a baseline US and three follow-up US after hematopoietic stem cell transplantation. Clinical criteria, including the European Society for Blood and Marrow Transplantation criteria, were used to diagnose veno-occlusive disease. We compared clinical factors and US parameters between the veno-occlusive disease and non-veno-occlusive disease groups. The diagnostic performance of US parameters for veno-occlusive disease was assessed by plotting receiver operating characteristic (ROC) curves. We describe temporal changes in US parameters before and after veno-occlusive disease diagnosis.

**Results:**

Among the 38 participants (mean age 10.7 years), eight developed veno-occlusive disease occurring 17.0 ± 5.2 days after hematopoietic stem cell transplantation. Liver stiffness, as measured by SWE (15.0 ± 6.2 kPa vs. 5.8 ± 1.8 kPa; *P*<0.001), and viscosity, as assessed with SWD (17.7 ± 3.1 m/s/kHz vs. 14.3 ± 2.8 m/s/kHz; *P*=0.015), were significantly higher in the veno-occlusive disease group compared to the non-veno-occlusive disease group at the time of diagnosis. Liver stiffness demonstrated the highest area under the ROC (AUROC) curves at 0.960, with an optimal predictive value of >6.5 kPa, resulting in sensitivity and specificity of 100% and 83.3%, respectively. Viscosity demonstrated an AUROC of 0.783, with an optimal cutoff value of 13.9 m/s/kHz for predicting veno-occlusive disease, with a sensitivity of 100% and specificity of 53.3%, respectively. Liver stiffness increased with disease severity and decreased during post-treatment follow-up.

**Conclusion:**

SWE may be a promising technique for early diagnosis and severity prediction of veno-occlusive disease. Furthermore, liver viscosity assessed by SWD may serve as an additional marker of veno-occlusive disease.

**Graphical abstract:**

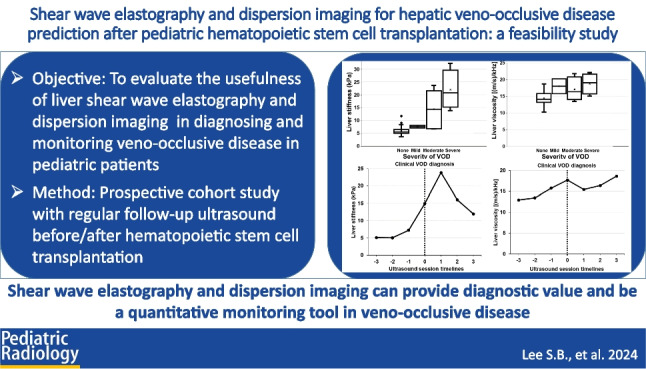

**Supplementary Information:**

The online version contains supplementary material available at 10.1007/s00247-024-05940-6.

## Introduction

Hepatic veno-occlusive disease is a drug-induced liver injury associated with chemotherapy regimens used in conditioning for hematopoietic stem cell transplantation. This injury disrupts venous outflow, leading to congestion and portal hypertension [1]. The reported incidence of veno-occlusive disease varies widely, ranging from 5% to 60%, depending on factors such as underlying diseases, conditioning regimens, types of transplantation, and prior therapies. Veno-occlusive disease is recognized as a life-threatening complication that can result in multiple organ failure [2–4].

The diagnosis of veno-occlusive disease primarily relies on clinical and biochemical parameters, such as hepatomegaly, weight gain, hyperbilirubinemia, and refractory thrombocytopenia [5]. Nevertheless, a non-invasive modality is warranted for diagnosing and assessing the severity since early diagnosis and treatment have been shown to improve the prognosis of veno-occlusive disease patients [6]. The most used imaging modality for veno-occlusive disease is grayscale and color Doppler ultrasound (US), which allows for evaluating parenchymal and hemodynamic changes [7–10]. However, the utility of this method may be limited, as specific US findings (such as the reversal of portal vein flow) only become apparent during the advanced stages of veno-occlusive disease [11].

Several imaging modalities combining grayscale, color Doppler, and elastography-derived quantified US information can aid early detection, treatment evaluation, and follow-up in pediatric veno-occlusive disease [12]. Quantitative elastography techniques include transient elastography and acoustic radiation force impulse techniques, including point shear wave elastography (SWE) and two-dimensional SWE [13]. The transient elastography technique might be a valuable tool for diagnosing veno-occlusive disease, but it has relatively high failure rates and small study populations in previous studies [14–16]. Acoustic radiation force impulse techniques use mechanical US impulses traveling perpendicular to compression waves, and they offer the advantage of appropriate sampling area placement with image acquisition.

SWE is a well-established technique used to detect and grade liver fibrosis in chronic liver diseases such as viral hepatitis or non-alcoholic fatty liver disease [17]. However, liver stiffness measured by SWE may be increased not solely due to fibrosis but to various other conditions, including inflammation, congestion, portal hypertension, and cholestasis [6]. Therefore, SWE can aid in the early detection of patients with suspected veno-occlusive disease [15–21]. Shear-wave dispersion (SWD) imaging reveals the shear wave slope, reflecting the viscosity associated with cellular inflammation, edema, and necrosis, providing a more comprehensive assessment of tissue characteristics compared to conventional US methods [22–24]. However, to the best of our knowledge, there are limited reports on the clinical utility of SWE in pediatric veno-occlusive disease, and no reports assessing the applicability of SWD to pediatric veno-occlusive disease.

Therefore, this study aimed to prospectively evaluate the utility of SWE and SWD imaging in diagnosing veno-occlusive disease and to assess whether quantitative US monitoring could be correlated with the clinical severity of hepatic veno-occlusive disease in pediatric patients who underwent hematopoietic stem cell transplantation.

## Materials and methods

This study complied with the Declaration of Helsinki and received approval from our institutional review board. Written informed consent was acquired from all the participants and their guardians.

### Participant selection

This prospective cohort study included consecutive pediatric patients who underwent hematopoietic stem cell transplantation at a single tertiary hospital from March 2021 to April 2022. Considering the documented prevalence of veno-occlusive disease in hematopoietic stem cell transplantation patients receiving myeloablative conditioning regimens at this institution, which exceeds 20% [25], we had planned to enroll 40 participants in this feasibility study.

Patients younger than 2 years were excluded because the SWE and SWD imaging require sufficient intercostal space to access the liver parenchyma. Other exclusion criteria included patients with pre-existing chronic liver disease at the pre-hematopoietic stem cell transplantation assessment to avoid potential bias, incomplete follow-up, and patients older than the age of 18 years. One patient was excluded because hematopoietic stem cell transplantation was canceled due to the worsening of the underlying disease after baseline US examination, and the other patient was excluded from the analysis due to being older than the age of 18 years.

### Multiparametric ultrasound examination

The study protocol consisted of four sessions: a baseline US before conditioning chemotherapy and three follow-up US after hematopoietic stem cell transplantation. The baseline US was performed 7 ± 3 days before hematopoietic stem cell transplantation (baseline session), followed by subsequent examinations at 7 ± 3 days (1st follow-up), 14 ± 3 days (2nd follow-up), and 28 ± 3 days (3rd follow-up) after hematopoietic stem cell transplantation. In addition, clinical data from all participants were collected on the date of the US examination.

The US examinations included grayscale, color Doppler, SWE, and SWD imaging techniques. All US examinations were conducted by an experienced pediatric radiologist (S.L. with 12 years of experience in pediatric US examination) using the same machine (Aplio i800, Canon Medical Systems, Otawara, Japan) with a 1–6 MHz convex transducer. The 1st and 2nd post-transplant follow-up US examinations were performed in a sterile room, considering the patient’s neutropenia, in contrast to other follow-up US examinations which were performed in the US room.

Following the criteria outlined in previous studies [9], the collected grayscale US findings encompassed: (1) liver size, determined by the craniocaudal length in the mid-axillary level sagittal scan (hepatomegaly defined as a size increase >1 cm compared to the baseline), (2) spleen size (splenomegaly defined as a size increase >1 cm compared to the baseline), (3) gallbladder wall thickness (gallbladder edema defined as thickness >6 mm), and (4) presence of ascites. Evaluated Doppler US findings encompassed (1) portal vein velocity (reversed or weak portal vein flow defined as velocity <10 cm/s), (2) hepatic vein flow phasicity (presence of monophasic flow), and (3) hepatic arterial resistive index (index >0.75).

SWE and SWD measurements were conducted in the intercostal view under gentle free-breath conditions. Quad-view mode, including SWE, SWD, propagation map, and corresponding grayscale map, can simultaneously depict four maps; we placed a 2 × 2 cm-sized sample box at a consistent depth below the liver capsule and obtained values within a uniform color-coded map with a parallel propagation area. The median stiffness (kPa) and viscosity (m/s/kHz) values, obtained from ten measurements on the SWE map and SWD map, were utilized in the analysis to yield reliable measurements [26].

For veno-occlusive disease patients, additional US follow-up examinations outside the study protocol were performed at the clinician’s request until there was no further clinical evidence of veno-occlusive disease. To assess serial changes in parameters before and after the diagnosis of veno-occlusive disease, we analyzed the changes in US data based on the time of diagnosis, including the additional US. In each patient, we reordered and named the US sessions as follows: the session just before diagnosis (− 1 session), the session at diagnosis (0 session), the first follow-up session after diagnosis (+ 1 session), and the second follow-up session after diagnosis (+ 2 session). The parameter values corresponding to each reordered US session were averaged across patients.

### Clinical assessment

The clinical diagnosis of veno-occlusive disease was based on the European Society for Blood and Marrow Transplantation criteria for pediatric patients [5]. Veno-occlusive disease was clinically diagnosed if two or more criteria were present: transfusion-refractory thrombocytopenia, weight gain (≥5% above baseline), bilirubin ≥2 mg/dL within 3 days, hepatomegaly, and ascites.

The severity of veno-occlusive disease was classified into three categories, ranging from mild to severe, based on clinical parameters assessed on the day of each US session. The severity of veno-occlusive disease by the European Society for Blood and Marrow Transplantation criteria included the following conditions: serological liver function abnormalities, such as increased transaminases to twice the normal range (mild) or less than five times (moderate), lasting days of refractory thrombocytopenia, hyperbilirubinemia, ascites, glomerular filtration rate, pulmonary, and coagulation status [5].

For veno-occlusive disease prophylaxis, all patients received a continuous infusion of lipo-prostaglandin E1 (Dongkook Pharm., Seoul, Korea) at a dose of 1 µg/kg per day with or without the addition of low-molecular-weight heparin (Handok Inc., Seoul, Korea) according to institutional guidelines for hematopoietic stem cell transplantation. Patients diagnosed with veno-occlusive disease were treated with defibrotide (Handok Inc.) until the resolution of veno-occlusive disease.

### Statistical analyses

The distribution of clinical variables was summarized, and a comparison was conducted between the clinical factors and US parameters of the veno-occlusive disease and non-veno-occlusive disease groups during the surveillance period following hematopoietic stem cell transplantation. Continuous variables between the groups were compared using either the independent *t*-test or the Wilcoxon rank-sum test. Categorical variables were analyzed using the Fisher’s exact test.

The diagnostic performance of the US parameters for detecting veno-occlusive disease was evaluated by constructing receiver operating characteristic (ROC) curves. Cutoff values for the US parameters were determined using the Youden J index to optimize sensitivity and specificity. A generalized estimating equation analysis was carried out to assess the predictors of veno-occlusive disease with the clinical and US parameters. This model accounted for the correlation between repeated measurements within an individual. Significant factors identified in the univariate analysis (those with *P*<0.20) were included in the multivariate analysis. In the multivariate analysis, statistical significance was defined as *P*<0.05. In addition, the descriptive analysis was only performed to compare the temporal changes in US data before/after clinical veno-occlusive disease diagnosis due to the limited number of patients in each session of veno-occlusive disease patients. All statistical analyses were conducted using SAS statistical software (SAS system for Windows, version 9.4; SAS Institute, Cary, NC).

## Results

### Participants characteristics

During the follow-up period after hematopoietic stem cell transplantation, 38 participants were assessed to determine the occurrence of veno-occlusive disease (Table 1). The average age of the participants was 10.7 ± 4.3 years. Eight patients developed veno-occlusive disease, with an average onset occurring 17.0 ± 5.2 days after hematopoietic stem cell transplantation. Among these, one patient had mild veno-occlusive disease, three had moderate veno-occlusive disease, and four had severe veno-occlusive disease based on clinical severity during the surveillance period.

**Table 1:** Participant characteristics

	VOD (*n*=8)	No VOD (*n*=30)	*P*-values^a^
Age (years, mean ± standard deviation)	9.8 ± 4.8	11.0 ± 4.3	0.488
Sex (female: male)	6:2	15:15	0.257
Disease			0.173
Acute myeloid leukemia	4 (50.0)^b^	5 (16.7)	
Acute lymphoblastic leukemia	1 (12.5)	4 (13.3)	
Solid tumors^c^	2 (25.0)	18 (60.0)	
Others^d^	1 (12.5)	3 (10.0)	
Types of HSCT			**0.008**
Allogenic			
Haploidentical	6 (75.0)	4 (13.3)	
Related	0 (0.0)	2 (6.7)	
Unrelated	0 (0.0)	5 (16.7)	
Autologous	2 (25.0)	19 (63.3)	
Conditioning regimens			0.131
Busulfan + Fludarabine	6 (75.0)	12 (40.0)	
Melphalan + Etoposide + Carboplatin	2 (25.0)	8 (26.7)	
Busulfan + Melphalan	0 (0.0)	10 (33.3)	
Initial presented VOD (days)	17.0 ± 5.2		
Clinical severity of VOD			
Mild	1 (12.5)		
Moderate	3 (37.5)		
Severe	4 (50.0)		

^a^Bold indicates statistical significance (*P*<0.05)

^b^All numbers in parentheses are percentages

^c^Solid tumors include osteosarcoma (*n*=9), Ewing sarcoma (*n*=7), medulloblastoma (*n*=3), and retinoblastoma (*n*=1)

^d^Others include lymphoma (*n*=3) and refractory cytopenia of childhood (*n*=1)

*HSCT* hematopoietic stem cell transplantation, *VOD* veno-occlusive disease

Regarding the underlying diseases and conditioning regimens, no significant differences were detected between the veno-occlusive disease and non-veno-occlusive disease groups. Haploidentical hematopoietic stem cell transplantation was more prevalent in the veno-occlusive disease group, while autologous hematopoietic stem cell transplantation was more common in the non-veno-occlusive disease group. Throughout the follow-up period, one patient (2.6%) in the veno-occlusive disease group experienced a mortality event associated with hematopoietic stem cell transplantation, with the cause determined to be septic shock and multi-organ failure. There were no cases of mortality related to veno-occlusive disease in our cohort.

### Comparison of veno-occlusive disease and non-veno-occlusive disease groups at the time of diagnosis

The US and biochemical parameters of both the veno-occlusive disease and non-veno-occlusive disease groups are presented in Table 2. In the comparison between the parameters at the time of veno-occlusive disease diagnosis in the veno-occlusive disease group and at the time of the second follow-up in the non-veno-occlusive disease group (which corresponds to the closest days to the average onset in the veno-occlusive disease group), the changes in liver and spleen size, gallbladder thickness, and the presence of ascites exhibited significantly higher values, portal vein velocity decreased, and monophasic flow of the hepatic vein in the veno-occlusive disease group. Liver stiffness and viscosity were significantly elevated in the veno-occlusive disease group compared to the non-veno-occlusive disease group.

**Table 2:** Comparison of parameters between patients with and without veno-occlusive disease

Parameters	VOD (*n*=8)	No VOD (*n*=30)	*P*-values^a^
Grayscale ultrasound			
Hepatomegaly	7 (87.5)^b^	5 (16.7)	**<0.001**
Liver size change (cm)	1.9 ± 1.1	0.2 ± 0.7	**<0.001**
Splenomegaly	3 (37.5)	4 (13.3)	0.122
Spleen size change (cm)	1.2 ± 1.4	0.2 ± 0.8	**0.043**
Gallbladder wall >6 mm	3 (37.5)	1 (3.3)	**0.006**
Gallbladder wall thickness (mm)	5.4 ± 3.4	2.0 ± 1.3	**0.001**
Ascites	6 (75.0)	3 (10.0)	**<0.001**
Color Doppler ultrasound			
Reversed or weak (<10 cm/s) portal flow	3 (37.5)	0 (0.0)	**<0.001**
Portal vein velocity (cm/s)	11.0 ± 9.4	19.9 ± 4.3	**0.006**
Monophasic flow in the hepatic vein	2 (25.0)	0 (0.0)	**0.005**
Hepatic artery resistive index ≥0.75	2 (25.0)	5 (16.7)	0.594
SWE and SWD			
Liver stiffness (kPa)	15.0 ± 6.2	5.8 ± 1.8	**<0.001**
Liver viscosity (m/s/kHz)	17.7 ± 3.1	14.3 ± 2.8	**0.015**
Biochemical parameters			
Aspartate aminotransferase (IU/L)	97.4 ± 163.6	38.0 ± 24.7	0.173
Alanine aminotransferase (IU/L)	54.8 ± 54.2	39.7 ± 39.8	0.430
Total bilirubin (mg/dL)	1.1 ± 0.5	0.6 ± 0.2	**<0.001**

^a^Bold indicates statistical significance (*P*<0.05)

^b^All numbers in parentheses are percentages

*SWD* shear-wave dispersion, *SWE* shear-wave elastography, *VOD* veno-occlusive disease

### Diagnostic performance of ultrasound parameters for veno-occlusive disease detection

The diagnostic performance of all US parameters was assessed in 152 US sessions involving 38 participants (four US sessions per participant) for veno-occlusive disease surveillance. This assessment included the area under the ROC curves (AUROCs), sensitivities, and specificities, which are presented in Table 3. Among these parameters, liver stiffness measured by SWE exhibited the highest AUROC of 0.960, and the optimal value for predicting veno-occlusive disease was >6.5 kPa, yielding a sensitivity and specificity of 100% and 83.3%, respectively. The second highest AUROC was observed for liver size change, with a value of 0.923 and a cutoff value of >0.9 cm, providing a sensitivity and specificity of 87.5% and 83.3%, respectively. Viscosity assessed through SWD imaging demonstrated an AUROC of 0.783, with an optimal cutoff value of 13.9 m/s/kHz for predicting veno-occlusive disease. The sensitivity and specificity corresponding to this cutoff value were 100% and 53.3%, respectively.

**Table 3:** Diagnostic performance of ultrasound parameters for indicating veno-occlusive disease

Parameters	AUROC	95% CI	*P*-values^a^	Cutoff	Sensitivity	Specificity
Grayscale ultrasound						
Liver size change (cm)	0.923	0.789–0.984	**<0.001**	>0.9	87.5	83.3
Spleen size change (cm)	0.735	0.567–0.865	**0.021**	>0.7	75.0	73.3
Gallbladder wall thickness (mm)	0.896	0.753–0.971	**<0.001**	>2.9	75.0	93.3
Ascites	0.825	0.667–0.929	**<0.001**	Present	75.0	90.0
Color Doppler ultrasound						
Portal vein velocity (cm/s)	0.821	0.663– 0.926	**0.002**	≤15.6	75.0	90.0
Monophasic flow in the hepatic vein	0.625	0.453–0.776	0.127	Present	25.0	100.0
Hepatic artery resistive index	0.623	0.451–0.775	0.333	>0.72	62.5	70.0
SWE and SWD						
Liver stiffness (kPa)	0.960	0.842–0.997	**<0.001**	>6.5	100	83.3
Liver viscosity (m/s/kHz)	0.783	0.620–0.900	**0.001**	>13.9	100	53.3

^a^Bold indicates statistical significance (*P*<0.05)

*AUROC* area under the receiver operating characteristic curve, *CI* confidence interval, *SWD* shear-wave dispersion, *SWE* shear-wave elastography

Considering the repeated measurements within an individual patient, several US parameters (the changes in liver and spleen size, ascites, portal vein velocity, and liver stiffness/viscosity) were significant parameters to assess the predictors of veno-occlusive disease in the univariate model. However, no biochemical parameters showed any critical indicator in this model (Table 4). In the multivariate model, an increase of 1 cm in liver size translated to a 2.2 times risk of veno-occlusive disease (*P*=0.009), whereas a 1 cm/s increase in portal vein velocity corresponded to a 0.9 times risk of veno-occlusive disease (*P*=0.008). However, the liver stiffness/viscosity was not a significant predictor for veno-occlusive disease in the multivariate model.

**Table 4:** Parameters related to the occurrence of veno-occlusive disease

**Parameters**	**Univariate analysis**		Multivariate analysis	
	OR (95% CI)	*P*-values^a^	Adjusted OR (95% CI)	*P*-values^a^
Grayscale ultrasound				
Liver size change (cm)	3.4 (1.9, 6.3)	**<0.001**	2.2 (1.2, 4.1)	**0.009**
Spleen size change (cm)	2.3 (1.1, 4.7)	**0.022**	1.4 (0.6, 2.9)	0.422
Gallbladder wall thickness (mm)	1.5 (1.0, 2.2)	**0.033**	1.2 (0.9, 1.5)	0.337
Ascites	8.5 (2.4, 30.1)	**0.001**	1.9 (0.5, 7.1)	0.330
Color Doppler ultrasound				
Portal vein velocity (cm/s)	0.8 (0.8, 0.9)	**<0.001**	0.9 (0.8, 1.0)	**0.008**
Monophasic flow in the hepatic vein	-	-		
Hepatic artery resistive index	-	-		
SWE and SWD				
Liver stiffness (kPa)	1.3 (1.2, 1.5)	**<0.001**	1.1 (0.9, 1.4)	0.183
Liver viscosity (m/s/kHz)	1.3 (1.2, 1.5)	**<0.001**	1.0 (0.8, 1.2)	0.994
Biochemical parameters				
Aspartate aminotransferase (IU/L)	1.0 (1.0, 1.0)	0.502		
Alanine aminotransferase (IU/L)	1.0 (1.0, 1.0)	0.414		
Total bilirubin (mg/dL)	3.3 (0.3, 36.8)	0.329		

^a^Bold indicates statistical significance (*P*<0.05) in the generalized estimating equation model

*CI* confidence interval, *OR* odds ratio, *SWD* shear-wave dispersion, *SWE* shear-wave elastography

### Quantitative monitoring using ultrasound parameters to clinical severity of veno-occlusive disease

**Fig. 1:**
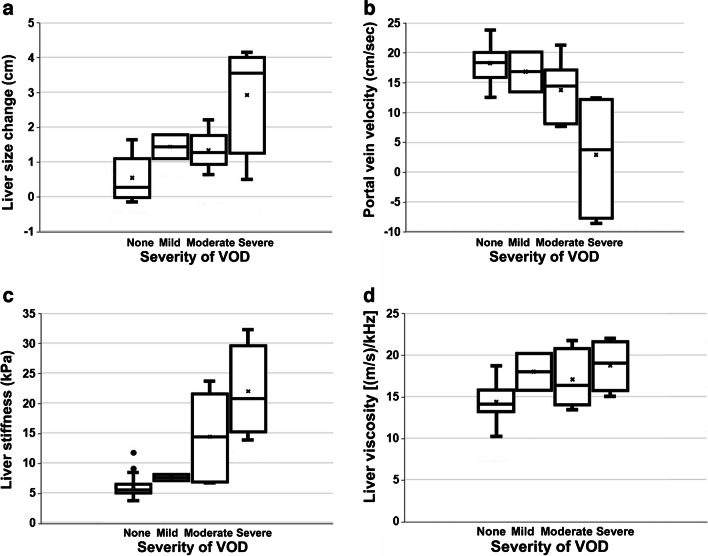
Ultrasound parameters according to severity of veno-occlusive disease. Thirty-two ultrasound sessions in eight patients with VOD exhibited the following distribution: none (*n*=20), mild (*n*=2), moderate (*n*=6), and severe (*n*=4). Box-and-whisker plots about ultrasound parameters showed the median, interquartile range, and outliers. **a**, **b** With increasing severity of VOD, liver size increased (**a**) and portal vein velocity decreased (**b**). **c**, **d** Shear wave elastography and dispersion imaging showed elevated liver stiffness in patients with moderate and severe veno-occlusive disease (**c**) and an increase in viscosity in all patients with veno-occlusive disease (**d**). *VOD* veno-occlusive disease

The clinical severity of veno-occlusive disease at each follow-up session based on the European Society for Blood and Marrow Transplantation criteria exhibited the following distribution (total 32 sessions; 8 patients undergoing four sessions each): none (*n*=20), mild (*n*=2), moderate (*n*=6), and severe (*n*=4). As severity increased, there was a corresponding increase in liver size and a decrease in portal vein velocity. Liver stiffness on SWE imaging and the liver viscosity on SWD imaging increased as clinical severity in veno-occlusive disease increased. In addition, biochemical parameters such as AST, ALT, and total bilirubin values showed an increasing trend as severity increased. A comparison of the parameters according to severity in each follow-up session of eight veno-occlusive disease patients is summarized in Fig. 1. The average values for each severity are presented in Supplementary Material 1.

**Fig. 2:**
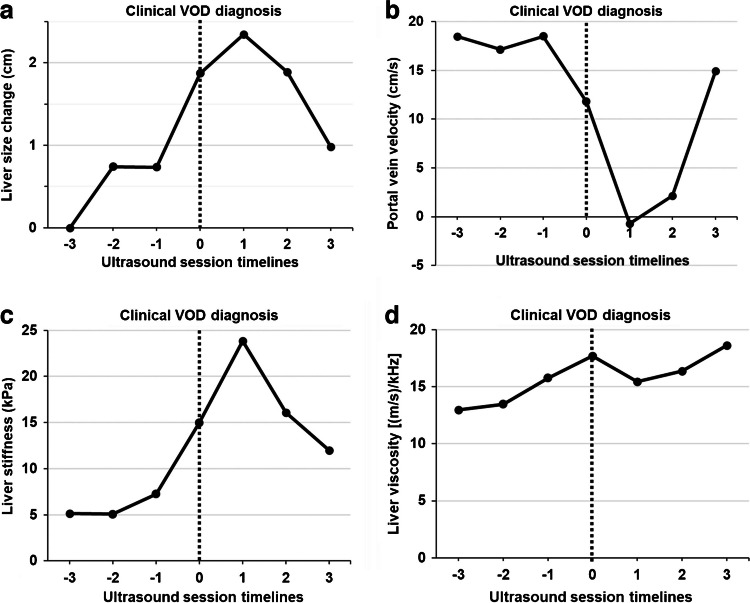
Changes in ultrasound parameters before and after diagnosis of veno-occlusive disease. Ultrasound session timelines were reordered based on the time of clinical diagnosis as follows: just before diagnosis (− 1 session), at diagnosis (0 session), and the first follow-up after diagnosis (+ 1 session). **a** Liver size increased more at clinical diagnosis of VOD than before diagnosis. **b** Portal venous flow decreased at the clinical diagnosis and markedly reduced at the first follow-up. **c** Liver stiffness revealed a slightly increased value before clinical diagnosis and increased at diagnosis and the first follow-up. **d** The liver viscosity was slightly increased before and at clinical diagnosis and remained at high values at the follow-up. *VOD *veno-occlusive disease

**Fig. 3:**
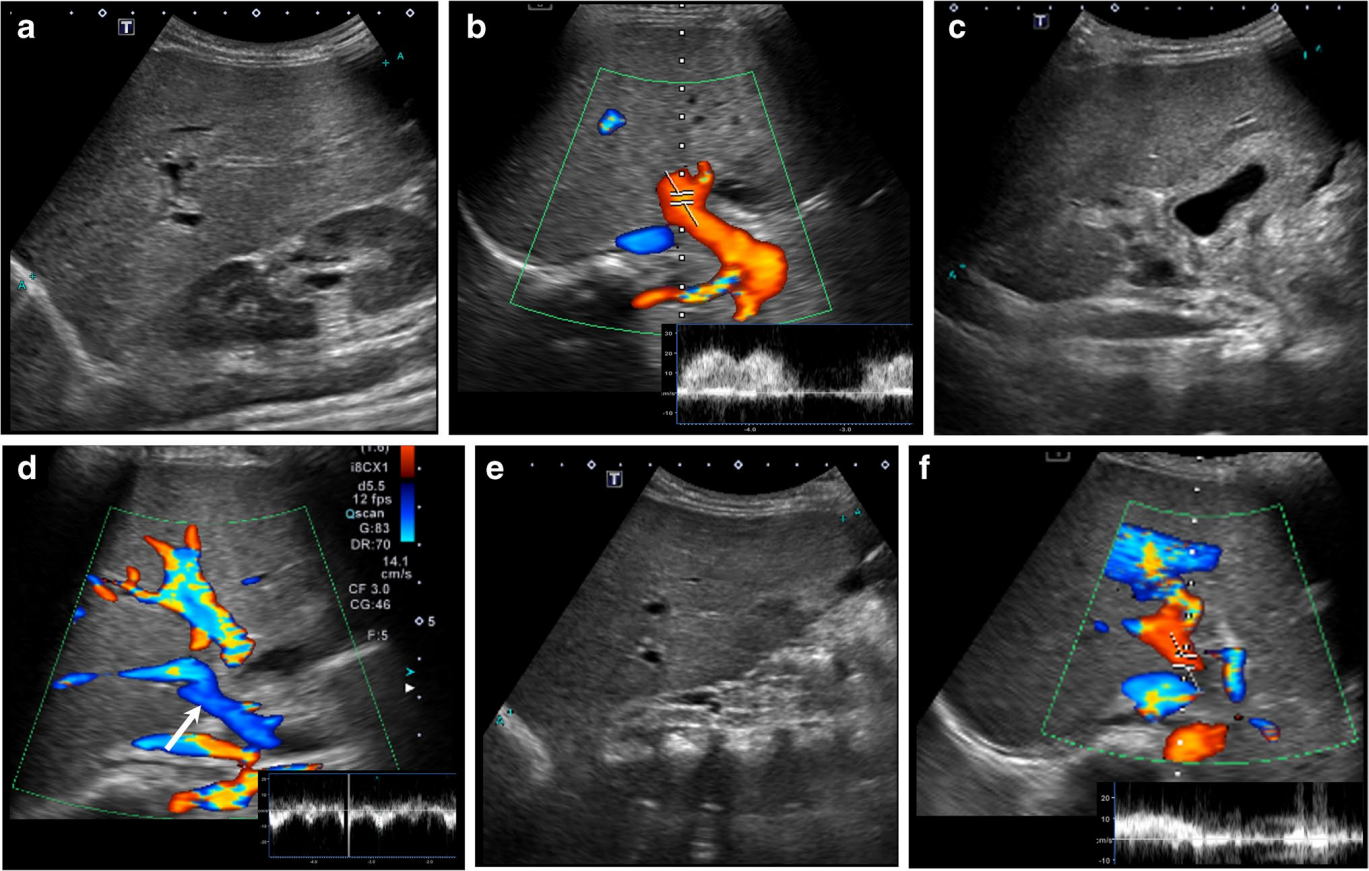
Sagittal ultrasound scans in a representative veno-occlusive disease (VOD) case.  A 6-year-old boy with acute myeloid leukemia underwent hematopoietic stem cell transplantation (HSCT). **a**, **b** Seven days after HSCT, the liver size increased to 13.4 cm (**a**), and the portal vein showed a typical velocity of 23.7 cm/s at the intercostal scan (**b**). Liver stiffness increased to 7.2 kPa and viscosity to 16.7 m/s/kHz at the intercostal scan. At that time, it did not meet the clinical diagnosis criteria for VOD. Three days later, the patient was clinically diagnosed with moderate veno-occlusive disease and treated with defibrotide. **c**, **d** Follow-up ultrasound on day 20 after HSCT revealed gallbladder wall edema (**c**) and reverse flow of the portal vein (*arrow* in **d**). Liver stiffness increased to 32.2 kPa and viscosity to 17.5 m/s/kHz. **e**, **f** A follow-up ultrasound on day 35 after HSCT showed a persistent liver size increase (**e**), but the portal vein recovered its flow direction and velocity (**f**). Liver stiffness decreased to 7.0 kPa, and viscosity remained at 16.9 m/s/kHz

In patients with veno-occlusive disease, additional US follow-up sessions extending the surveillance period were performed in five of the eight veno-occlusive disease patients: one US session in two patients, two US in one patient, and three US in two patients. The temporal changes of the parameters across each patient before and after veno-occlusive disease diagnosis are shown in Fig. 2 and Supplementary Material 2. Liver size and gallbladder wall thickness increased at the time of diagnosis but decreased at the second follow-up. Liver stiffness increased just before veno-occlusive disease diagnosis and dropped at the subsequent follow-up. Portal venous flow velocity was slightly reduced at diagnosis, and the reverse was identified at the ensuing follow-up immediately after diagnosis. In addition, liver viscosity, ascites, and bilirubin increased from the time of diagnosis and remained at similar levels. Surveillance US sessions just before clinical diagnosis revealed liver stiffness higher than our cutoff value of 6.5 kPa in four veno-occlusive disease cases (50.0%). An early detected case of veno-occlusive disease using US parameters before a clinical diagnosis is shown in Fig. 3.

## Discussion

This study shows that SWE and SWD can provide diagnostic evidence for veno-occlusive disease and serve as quantitative monitoring tools in pediatric patients who undergo hematopoietic stem cell transplantation. The increase in liver stiffness and viscosity emerged as significant factors for veno-occlusive disease diagnosis. Liver stiffness showed the highest diagnostic performance among US parameters and demonstrated an increase as the severity of veno-occlusive disease increased. Liver stiffness increased at the time of diagnosis and subsequently decreased during post-treatment follow-up. Additionally, liver stiffness displayed an early rise in four patients before clinical diagnosis among all patients with veno-occlusive disease.

The traditional diagnostic criteria for veno-occlusive disease have relied on clinical parameters, such as elevated bilirubin levels and weight gain [27, 28]. However, these criteria have faced criticism due to the challenge of identifying patients with veno-occlusive disease who require early intervention [29, 30]. Consequently, the European Bone and Marrow Transplantation Society criteria have incorporated recommendations for diagnostic imaging instead of relying solely on partly subjective assessments of parameters such as pain and hepatomegaly [30]. This study demonstrates that hepatomegaly, ascites, and decreased portal venous velocity can aid in the diagnosis of veno-occlusive disease, aligning with studies that assessed veno-occlusive disease using US [9, 10]. Although a reduction or reversal of portal venous flow observed in severe veno-occlusive disease patients was considered more specific for veno-occlusive disease, clinicians should not delay the diagnosis and initiation of treatment even if portal flow reversal is absent [27]. Early diagnosis and accurate severity classification of veno-occlusive disease could facilitate the prompt initiation of treatment at an early stage, potentially correlating with improved outcomes for patients undergoing hematopoietic stem cell transplantation [31, 32].

The rationale for using liver stiffness and viscosity as early detectors of veno-occlusive disease is related to its pathogenesis [15]. The conditioning regimens can induce toxic damage to sinusoidal endothelial cells, resulting in local inflammation and migration of blood cells into the space of Disse, causing obstruction of the sinusoidal microcirculation, which can result in sinusoidal portal hypertension [3, 15, 19]. A recent retrospective study utilized SWE to diagnose veno-occlusive disease in pediatric patients [21], and found that a liver stiffness of 7.2 kPa or greater had a sensitivity of 84.2% and a specificity of 93.3% in differentiating veno-occlusive disease from non-veno-occlusive disease. However, due to its retrospective design, the study had inherent limitations, such as the absence of baseline SWE values before hematopoietic stem cell transplantation and irregular follow-up periods. The prospective nature of our study allowed us to measure baseline liver stiffness before hematopoietic stem cell transplantation, and we performed regular follow-ups for each patient after hematopoietic stem cell transplantation, regardless of symptoms. Therefore, it was possible to evaluate the changes in the patient’s liver stiffness values according to the progression of veno-occlusive disease and suggest a cutoff value that could be used to diagnose veno-occlusive disease. The average liver stiffness of all the patients in baseline US was 5.6 kPa, and liver viscosity was 13.6 m/s/kHz; in the non-veno-occlusive disease group, the average value during all the sessions was 5.7 kPa and 13.8 m/s/kHz, which were consistent with those of a previous report [30]. This study demonstrated that the average value of liver stiffness in the veno-occlusive disease group was 15.0 kPa, and liver viscosity was 17.7 m/s//kHz at initial diagnosis. The sensitivity and specificity of predicting veno-occlusive disease when liver stiffness exceeded 6.5 kPa were 100% and 83.3%.

Moreover, liver stiffness increased according to disease severity (mild; 7.5 kPa, moderate; 14.4 kPa, severe, 21.8 kPa). Previous case series reported that SWE showed increased liver stiffness before clinical diagnosis and reduced within 2–4 weeks in specifically treated patients [15, 16]. This study also demonstrated high stiffness based on cutoff values before clinical diagnosis in veno-occlusive disease patients and a subsequent decrease in stiffness after treatment with defibrotide. Therefore, liver stiffness could serve as a discriminator for recognizing the progression or improvement of veno-occlusive disease and for the early detection of veno-occlusive disease during initial screenings. Regarding the histological changes in veno-occlusive disease, fibrosis is not observed during the acute phase of toxic injury. Instead, there is a development of dense perivascular fibrosis radiating into the parenchyma as the changes persist over weeks to months [33]. Therefore, the elevated liver stiffness observed in the acute phase of veno-occlusive disease and the subsequent decrease after treatment might be attributed to changes induced by inflammation, congestion, or portal hypertension rather than fibrosis.

According to a previous report and guidelines [34, 35], liver stiffness on US elastography varies considerably depending on different machines, transducers, and acquisition depths. US elastography using various transducers/machines makes it difficult to generalize the diagnostic criteria because it is difficult to set a cutoff value. However, for pediatric hematologic oncology patients scheduled to receive hematopoietic stem cell transplantation, follow-up monitoring while measuring the stiffness value using the same ultrasound equipment will be helpful in diagnosis and veno-occlusive disease monitoring. Liver viscosity at SWD was also significantly higher in the veno-occlusive disease group than in the non-veno-occlusive disease group and was significantly associated with the occurrence of veno-occlusive disease. However, it did not increase before clinical diagnosis, exhibit an immediate decrease after treatment, or greatly vary with disease severity. Similarly, prior reports did not identify viscosity as a reliable predictor of disease activity [36, 37]. In addition, the multivariate model did not identify any predictors for the development of veno-occlusive disease on SWE/SWD imaging when all other risk factors were included. Therefore, we should also consider conventional diagnostic findings in veno-occlusive disease using gray-scale and color Doppler during veno-occlusive disease surveillance.

This study has several limitations. First, selection bias could be an inherent problem, as this study was conducted at a single tertiary hospital. Additionally, US examinations were performed by a single radiologist. Therefore, interobserver agreement could not be assessed, and the generalizability of the results is limited. However, we could identify change during the longitudinal follow-up because it was a prospective study performed by a single radiologist on the same machine and consistently following the protocol. Additional multicenter future studies are warranted to identify the role of SWE and SWD in the preclinical diagnosis of veno-occlusive disease. Second, the number of patients with veno-occlusive disease was small. However, the incidence of veno-occlusive disease in our study was 20.5%, consistent with other reports on pediatric patients [25]. Thirdly, due to the inclusion of young children, we conducted under gentle free breath conditions. A prior study demonstrated that pediatric liver SWE outcomes were comparable under free-breathing and breath-holding conditions [38]. Finally, the time interval was arbitrarily determined for the three US evaluations after hematopoietic stem cell transplantation. Since evidence for the standard US schedule of pediatric patients receiving hematopoietic stem cell transplantation has not yet been established, the schedule currently used in our hospital (Seoul National University Hospital) was applied. These intervals could mask actual short-term interval changes. However, performing a daily US examination on patients in a sterile room may not be practical.

In conclusion, this prospective cohort study conducted in pediatric patients, mirroring real hematopoietic stem cell transplantation practice, suggests that SWE could be a promising technique for early diagnosis and severity prediction of veno-occlusive disease. Additionally, it aids in evaluating the treatment response in pediatric patients with veno-occlusive disease. Furthermore, liver viscosity assessed by SWD may serve as an additional marker for veno-occlusive disease diagnosis, although it was not found to be correlated with disease severity.

### Electronic Supplementary material

Below is the link to the electronic supplementary material.

Supplementary Material 1 (DOCX 24.3 KB)

## Data Availability

The datasets generated or analyzed during the study are available from the corresponding author on reasonable request.
