# Correction to: Shear wave elastography and dispersion imaging for hepatic veno‑occlusive disease prediction after pediatric hematopoietic stem cell transplantation: a feasibility study

**DOI:** 10.1007/s00247-024-05963-z

**Published:** 2024-05-30

**Authors:** Seul Bi Lee, Seunghyun Lee, Yeon Jin Cho, Young Hun Choi, Jung‑Eun Cheon, Kyung Taek Hong, Jung Yun Choi, Hyoung Jin Kang

**Affiliations:** 1 Department of Radiology, Seoul National University Hospital, 101 Daehak‑ro, Jongno‑gu, Seoul, 03080 Republic of Korea; 2 Department of Radiology, Seoul National University College of Medicine, Seoul, Republic of Korea; 3 Institute of Radiation Medicine, Seoul National University Medical Research Center, Seoul, Republic of Korea; 4 Department of Pediatrics, Seoul National University Hospital, Seoul, Republic of Korea; 5 Department of Pediatrics, Seoul National University College of Medicine, Seoul, Republic of Korea; 6 Seoul National University Cancer Research Institute, Seoul, Republic of Korea; 7 Wide River Institute of Immunology, Seoul National University, Gangwon‑do, Hongcheon‑gun, Republic of Korea

**Correction to** **:** **Pediatric Radiology (2024)**

10.1007/s00247-024-05940-6

The original article contains some errors. Below are the corrections.

- 1. Under the heading “Multiparametric ultrasound examination” it is stated, “The baseline US was performed 7 ± 3 days before hematopoietic stem cell transplantation (baseline session), followed by subsequent examinations at s7 ± 3 days (1st follow-up), 14 ± 3 days (2nd follow-up), and 28 ± 3 days (3rd follow-up) after hematopoietic stem cell transplantation.”
  This is an error, and the sentence should read, “The baseline US was performed 7 ± 3 days before hematopoietic stem cell transplantation (baseline session), followed by subsequent examinations at 7 ± 3 days (1st follow-up), 14 ± 3 days (2nd follow-up), and 28 ± 3 days (3rd follow-up) after hematopoietic stem cell transplantation.”
- 2. In the original published online version, there was an error in the uploading of Figure 3. The figure has since been updated.
- 3. The original published online version included the tracked version of the supplementary file. This has been updated.

The original article has been corrected.

