# Supplementary material for: Shear wave elastography and dispersion imaging for hepatic veno-occlusive disease prediction after pediatric hematopoietic stem cell transplantation: a feasibility study
Source: Pediatr Radiol. 2024 May 22;54(7):1144–55. doi: 10.1007/s00247-024-05940-6 (PMC11182801; doi:10.1007/s00247-024-05940-6)
Supplement: Supplementary file 1 — (DOCX 24.3 KB) [file 247_2024_5940_MOESM1_ESM.docx]

**Supplementary material 1. A comparison of the parameters according to severity in eight veno-occlusive disease patients**

| Parameter | None (*n*=20)^a^ | Mild (*n*=2) | Moderate (*n*=6) | Severe (*n*=4) |
| --- | --- | --- | --- | --- |
| Gray-scale ultrasound |  |  |  |  |
| Hepatomegaly | 7 (35.0)^b^ | 2 (100.0) | 5 (83.3) | 3 (75.0) |
| Liver size change (cm) | 0.6±0.6 | 1.4±0.5 | 1.3±0.5 | 2.9±1.7 |
| Splenomegaly | 4 (20.0) | 1 (50.0) | 1 (16.7) | 2 (50.0) |
| Spleen size change (cm) | 0.3±0.8 | 1.0±1.2 | 0.7±0.4 | 1.8±1.8 |
| Gallbladder wall > 6mm | 1 (5.0) | 0 (0.0) | 3 (50.0) | 2 (50.0) |
| Gallbladder wall thickness (mm) | 2.3±1.7 | 5.2±0.4 | 6.1±3.6 | 5.2±3.1 |
| Ascites | 2 (10.0) | 1 (50.0) | 5 (83.3) | 3 (75.0) |
| Color Doppler ultrasound |  |  |  |  |
| Reversed portal flow | 0 (0.0) | 0 (0.0) | 2 (33.3) | 2 (50.0) |
| Portal vein velocity (cm/sec) | 18.0±3.0 | 16.8±4.6 | 11.1±10.1 | 9.3±3.7 |
| Monophasic hepatic vein flow | 1 (5.0) | 0 (0.0) | 0 (0.0) | 2 (50.0) |
| Hepatic artery RI ≥0.75 | 7 (35.0) | 1 (50.0) | 2 (33.3) | 0 (0.0) |
| SWE and SWD |  |  |  |  |
| Liver stiffness (kPa) | 6.0±1.8 | 7.5±0.8 | 14.4±7.0 | 21.8±7.7 |
| Liver viscosity (m/s/kHz) | 14.3±2.3 | 17.9±3.1 | 17.1±3.4 | 18.7±3.1 |
| Biochemical Parameters |  |  |  |  |
| Aspartate aminotransferase (IU/L) | 25.5±6.5 | 34.0±11.3 | 39.0±15.0 | 171.5±221.1 |
| Alanine aminotransferase (IU/L) | 24.1±8.2 | 14.5±0.7 | 40.7±18.4 | 71.8±74.1 |
| Total bilirubin (mg/dL) | 0.9±0.4 | 1.1±0.6 | 1.1±0.5 | 1.3±0.5 |

^a^The total number of ultrasound sessions

^b^All numbers in parentheses are percentages

*RI* resistive index, *SWD* shear-wave dispersion, *SWE* shear-wave elastography

**Supplementary material 2. Changes in ultrasound parameters before and after diagnosis of veno-occlusive disease**

| Parameter | Just before diagnosis (*n*=8)^a^ | At the time of diagnosis (*n*=8) | 1^st^ follow-up after diagnosis (*n*=6) | 2^nd^ follow-up after diagnosis *(n*=4) |
| --- | --- | --- | --- | --- |
| Gray-scale ultrasound |  |  |  |  |
| Hepatomegaly | 3 (37.5)^b^ | 6 (75.0) | 5 (83.3) | 3 (75.0) |
| Liver size change (cm) | 0.7±0.6 | 1.9±1.1 | 2.3±1.3 | 1.9±0.9 |
| Splenomegaly | 3 (37.5) | 3 (37.5) | 3 (50.0) | 4 (100.0) |
| Spleen size change (cm) | 0.4±1.0 | 1.2±1.4 | 1.5±1.3 | 2.1±0.9 |
| Gallbladder wall > 6 mm | 1 (12.5) | 3 (37.5) | 3 (50.0) | 0 (0.0) |
| Gallbladder wall thickness (mm) | 2.9±2.6 | 5.4±3.4 | 5.7±2.0 | 3.7±1.6 |
| Ascites | 1 (12.5) | 6 (75.0) | 5 (83.3) | 3 (75.0) |
| Color Doppler ultrasound |  |  |  |  |
| Reversed portal flow | 0 (0.0) | 1 (12.5) | 5 (83.3) | 2 (50.0) |
| Portal vein velocity (cm/s) | 18.5±3.6 | 11.8±8.2 | -0.7±13.4 | 2.2±17.6 |
| Monophasic hepatic vein flow | 1 (12.5) | 2 (25.0) | 1 (16.7) | 0 (0.0) |
| Hepatic artery RI ≥ 0.75 | 3 (37.5) | 2 (25.0) | 1 (16.7) | 0 (0.0) |
| SWE and SWD |  |  |  |  |
| Liver stiffness (kPa) | 7.3±2.3 | 15.0±6.2 | 23.9±11.5 | 16.1±11.7 |
| Liver viscosity (m/s/kHz) | 15.8±2.2 | 17.7±3.1 | 15.4±3.5 | 16.4±3.4 |
| Biochemical Parameters |  |  |  |  |
| Aspartate aminotransferase (IU/L) | 26.0±5.4 | 97.4±163.6 | 126.5±94.8 | 69.8±64.1 |
| Alanine aminotransferase (IU/L) | 22.8±6.5 | 54.8±54.2 | 85.2±77.2 | 58.8±44.7 |
| Total bilirubin (mg/dL) | 1.0±0.4 | 1.1±0.5 | 1.3±0.5 | 1.3 ±0.3 |

^a^The total number of ultrasound sessions

^b^All numbers in parentheses are percentages

*RI* resistive index, *SWD* shear-wave dispersion, *SWE* shear-wave elastography
